# Virological outcome among HIV-1 infected patients on first-line antiretroviral treatment in semi-rural HIV clinics in Togo

**DOI:** 10.1186/s12981-015-0082-7

**Published:** 2015-11-27

**Authors:** Abla A. Konou, Mounerou Salou, Nicole Vidal, Pascal Kodah, Damobé Kombate, Pyabalo Kpanla, Tchabia Nabroulaba, Djifa Nyametso, Assétina Singo-Tokofaï, Palokinam Pitche, Eric Delaporte, Mireille Prince-David, Martine Peeters, Anoumou Y. Dagnra

**Affiliations:** Laboratoire de Biologie Moléculaire et d’Immunologie (BIOLIM/FSS/UL), Université de Lomé, Lomé, Togo; Département des sciences fondamentales et biologiques, Faculté des Sciences de la Santé, Université de Lomé, Lomé, Togo; UMI 233, Institut de Recherche pour le Développement (IRD)/INSERM U1175, Université de Montpellier, Montpellier, France; Centre Médico-Social (CMS) Kouvé, Kouvé, Togo; Hopital Général d’Aného, Aného, Togo; Centre Hospitalier Préfectoral (CHP) Kpalimé, Kpalimé, Togo; Centre Hospitalier Universitaire (CHU) Kara, Kara, Togo; Centre Hospitalier Régional (CHR) Atakpamé, Atakpamé, Togo; Programme National de Lutte contre le Sida (PNLS), Lomé, Togo; Conseil National de Lutte contre le Sida (CNLS), Lomé, Togo; 08 B.P. 8742, Lomé 08, Togo

**Keywords:** HIV, Antiretroviral treatment, Drug resistance, Semi-rural, Public health, Togo, Africa

## Abstract

**Background:**

Access to antiretroviral treatment (ART) in resource-limited countries has increased significantly but scaling-up ART into semi-rural and rural areas is more recent. Information on treatment outcome in such areas is still very limited notably due to additional difficulties to manage ART in these areas.

**Results:**

387 HIV-1 infected adults (≥18 years) were consecutively enrolled when attending healthcare services for their routine medical visit at 12 or 24 months on first-line ART in five HIV care centers (four semi-rural and one rural). Among them, 102 patients were on first-line ART for 12 ± 2 months (M12) and 285 for 24 ± 2 months (M24). Virological failure was observed in 70 (18.1 %) patients ranging from 13.9 to 31.6 % at M12 and from 8.1 to 22.4 % at M24 across the different sites. For 67/70 patients, sequencing was successful and drug resistance mutations were observed in 65 (97 %). The global prevalence of drug resistance in the study population was thus at least 16.8 % (65/387). Moreover, 32 (8.3 %) and 27 (6.9 %) patients were either on a completely ineffective ART regime or with only a single drug active. Several patients accumulated high numbers of mutations and developed also cross-resistance to abacavir, didanosine or the new NNRTI drugs like etravirine and rilpivirine.

**Conclusion:**

The observations on ART treatment outcome from ART clinics in semi-rural areas are close to previous observations in Lomé, the capital city suggesting that national ART-programme management plays a role in treatment outcome.

## Findings

### Background

Scale-up of antiretroviral treatment (ART) programs in resource-limited countries was possible because standardized first and second line antiretroviral (ARV) combinations and clinical and/or immunological criteria to start and monitor ART were used as recommended by WHO [1]. However, heterogeneous treatment outcomes have been observed in the national ART programs from different countries, most likely related to ART-programme management [2]. As such, virological failure can range from less than 3 % to more than 20 % in patients on ART for 12 or 24 months [2–4].

In Togo, a country of six million inhabitants in West Africa, scaling-up of ART started in 2007 in Lomé, the capital city, and has expanded to semi-rural areas in 2008. In 2013, almost 50 % of patients who were in need for ART according to WHO guidelines from 2010 (CD4 count <350) were receiving ART [5]. Previous studies in ART clinics in Lomé showed high virological failure related to ARV drug resistance, i.e. in 13 to 25 % of the patients receiving ART for 12 or 24 months [2, 6]. Given the additional difficulties to manage ART in these areas (distances to clinics, scarce human resources, drug stock-outs, etc.) together with high rates of ARV drug resistance in the capital city, it was important to evaluate also virological outcome and emergence of drug resistance in ART clinics located in semi-urban and rural areas in Togo.

### Methods

#### Study sites and population

**Fig. 1:**
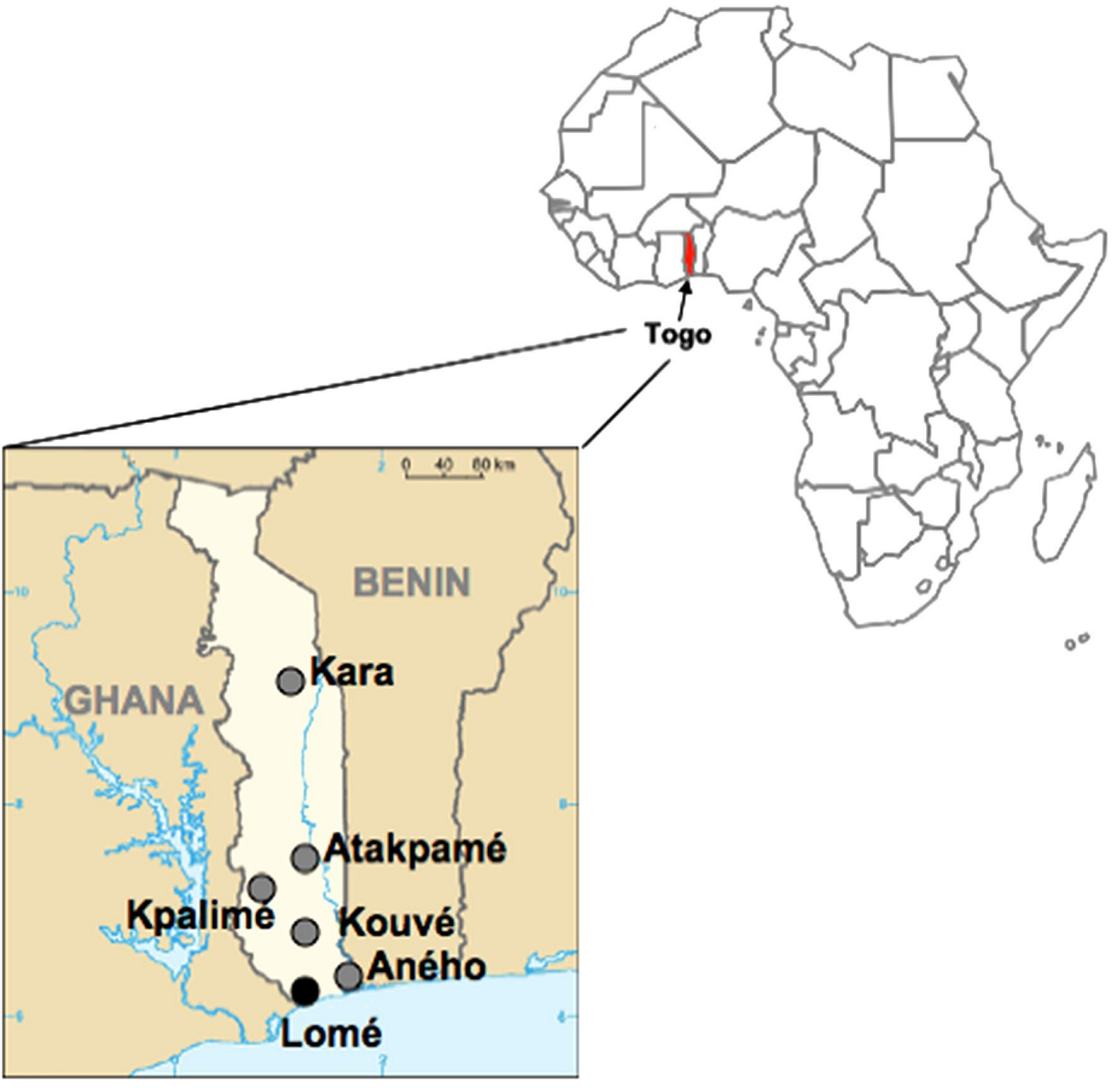
Togo map indicating locations of the healthcare centers where patients were enrolled. The sites where samples were collected for this study are indicated with *grey full circles*, and the name of the corresponding city at the *right*. Lomé, the capital city, is indicated with a *black full circle*

A cross-sectional study was conducted in 2012 between January and July in five HIV care centers that administer ARV drugs and monitor treatment. They were located in four semi-rural cities: Aného (AN), Kpalimé (KP), Atakpamé (AT) and Kara (KA) at respectively 60, 120, 160 and 410 km from Lomé, the capital city, and in one rural city, Kouvé (KO) at 70 km distance from Lomé (Fig. 1). HIV-1 infected adults (≥18 years) were consecutively enrolled when attending clinics for their routine medical visit at 12 ± 2 months or 24 ± 2 months on first-line ART. This study was approved by the National Ethics Committee (nº751/2014/MS/CAB/DGS/DPLET/CBRS). Informed consent was obtained for each participating patient. Questionnaires were used to collect epidemiological and demographic information and ART history was obtained from on-site medical records. Whole blood was drawn and plasma was separated by centrifugation. Plasma aliquots were stored at −20 °C for maximum 1 week on site and were subsequently transported by road in a cool box to the Laboratoire de Biologie Moléculaire et d’Immunologie (BIOLIM/FSS-UL) where they were stored at −80 °C until use.

#### Virological analyses

HIV-1 viral load (VL) was determined with EasyQ HIV assay (Biomerieux, Capronne, France) or RealTime m2000rt (Abbott Pack, IL, USA) in Lomé (BIOLIM/FSS-UL). According to WHO recommendations, genotypic drug resistance testing was done in a WHO accredited laboratory (IRD, Montpellier, France) on patients with VL ≥1000 copies/ml. Protease and partial Reverse Transcriptase (RT) were amplified with the in-house protocol from the Agence Nationale de Recherche sur le Sida et les Hépatites en France (ANRS) [2, 7]. Drug resistance mutations (DRM) were identified using the ANRS interpretation algorithm, version 24 [7]. The newly reported sequences are available in GenBank under the following accession numbers: KR047793–KR047859.

### Results

A total of 387 patients were consecutively enrolled during their follow-up visit at 12 ± 2 (M12, n = 102) or 24 ± 2 (M24, n = 285) months on ART. Table 1 shows patients characteristics at each site. Overall, more women were enrolled than men: 84/102 (82.4 %) at M12 and 205/285 (71.9 %) at M24. The median age of patients was 36 (IQR 31–42) and 39 years (IQR 33–45) at M12 and M24, respectively. More than 97 % (277/285) of patients have been exposed to the following drugs in their first line regimen: stavudine (d4T) and/or zidovudine (AZT) plus lamivudine (3TC) plus nevirapine (NVP) and/or efavirenz (EFV). For 302 (78 %) patients, d4T was replaced by AZT because national guidelines were changed. Only eight (2.8 %) patients switched to tenofovir (TDF) instead of AZT or d4T. At ART initiation the overall median CD4 count/mm^3^ was 176 (IQR 86–261) and 152 (IQR 88–219) for the patients who were on ART for 12 ± 2 and 24 ± 2 months, respectively. Overall, 153/373 (41.1 %) and 220/373 (58.9 %) of the patients were in WHO stages 1 or 2 and WHO stages 3 or 4, respectively at ART start. However, CD4 counts and WHO stages at ART initiation could vary across the different sites (Table 1).

**Table 1:** Characteristics of patients with 12 (M12) or 24 (M24) months ART experience

Characteristics	M12					M24					
	Aného (AN)	Kpalimé (KP)	Atakpamé (AT)	Kara (KA)	Total	Aného (AN)	Kouvé (KO)	Kpalimé (KP)	Atakpamé (AT)	Kara (KA)	Total
Number of patients (n)	19	14	26	43	102	58	103	37	50	37	285
Women (%)	17 (89.5 %)	13 (92.8 %)	20 (76.9 %)	34 (79.1 %)	84 (82.4 %)	42 (72.4 %)	74 (71.9 %)	26 (70.3 %)	39 (78.0 %)	24 (64.9 %)	205 (71.9 %)
Median age years (IQR)	42 (35–51)	38 (36–49)	35 (33–42)	36 (31–40)	36 (31–42)	40 (35–44)	38 (32–45)	40 (34–46)	36 (30–46)	37 (33–42)	39 (33–45)
WHO stages											
1/2	2/19 (10.5 %)	9/14 (64.3 %)	15/24 (62.5 %)	20/42 (47.6 %)	46/99 (46.4 %)	13/58 (22.4 %)	24/102 (23.5 %)	18/34 (52.9 %)	39/43 (90.7 %)	13/37 (35.1 %)	107/274 (39.1 %)
3/4	17/19 (89.5 %)	5/14 (35.7 %)	9/24 (37.5 %)	22/42 (52.4 %)	53/99 (53.6 %)	45/58 (77.6 %)	78/102 (76.5 %)	16/34 (47.1 %)	4/43 (9.3 %)	24/37 (64.9 %)	167/274 (60.9 %)
CD4 counts available at baseline (n)	18 (94.7 %)	14 (100 %)	26 (100 %)	43 (100 %)	101 (99.0 %)	56/58 (96.6 %)	103/103 (100 %)	31/37 (83.8 %)	42/50 (84 %)	35/37 (94.6 %)	667/285 (93.7 %)
Median CD4 counts at baseline (IQR)	107 (86–181)	109 (71–235)	197 (111–277)	202 (101–274)	176 (86–261)	134 (110–210)	154 (75–232)	120 (80–158)	201 (167–242)	135 (106–185)	152 (88–219)
First line drugs n (%)											
AZT-3TC-EFV	1 (5.3 %)	–	1 (3.8 %)	1 (2.3 %)	3 (2.9 %)	1 (1.7 %)	6 (5.8 %)	2 (5.4 %)	10 (20.0 %)	3 (8.1 %)	23 (8.1 %)
AZT-3TC-NVP	1 (5.3 %)	–		10 (23.3 %)	11 (10.8 %)	7 (12.1 %)	–	–	6 (12.0 %)	–	13 (4.6 %)
AZT-3TC-NVP/EFV	–	–	–	–	–	–	2 (1.9 %)	–	–	–	2 (0.7 %)
D4T-3TC-NVP	1 (5.3 %)	5 (35.7 %)	7 (26.9 %)	2 (4.7 %)	15 (14.7 %)	4 (6.9 %)	3 (2.9 %)	–	1 (2.0 %)	1 (2.7 %)	9 (3.2 %)
D4T/AZT-3TC-NVP	16 (84.2 %)	7 (20.0)	15 (57.7 %)	28 (65.1 %)	66 (64.7 %)	43 (75.8 %)	86 (83.5 %)	32 (86.5 %)	32 (64.0 %)	33 (89.2 %)	226 (79.3 %)
D4T/AZT-3TC-NVP/EFV	–	2 (10.0 %)	1 (3.8 %)		3 (2.9 %)	3 (5.2 %)	1 (0.98 %)	3 (8.1 %)	–	–	7 (2.5 %)
D4T/AZT/TDF-3TC-NVP/EFV	–	–	1 (3.8 %)	1 (2.3 %)	2 (1.9 %)	–	2 (1.9 %)	–	–	–	2 (0.7 %)
TDF-3TC-AZT	–	–	–	–	–	–	1 (0.98 %)	–	–	–	1 (0.3 %)
TDF-3TC-NVP	–	–	1 (3.8 %)	–	1 (0.99 %)	–	–	–	–	–	–
AZT/TDF-3TC-NVP/EFV	–	–	–	1 (2.3 %)	1 (0.99 %)	–	–	–	–	–	–
D4T/AZT/TDF-3TC-EFV	–	–	–	–	–	–	1 (0.98 %)	–	–	–	1 (0.3 %)
AZT/TDF-3TC-EFV	–	–	–	–	–	–	1 (0.98 %)	–	–	–	1 (0.3 %)
VL >1000 copies/ml n (%)	6/19 (31.6 %)	3/14 (23.1 %)	5/26 (19.2 %)	6/43 (13.9 %)	20/102 (19.6 %)	13/58 (22.4 %)	14/103 (13.6 %)	11/37 (29.7 %)	9/50 (18.0 %)	3/37 (8.1 %)	50/285 (17.5 %)
Obtained pol sequences (n/n tested)	6/6	3/3	5/5	6/6	20/20	12/13	14/14	10/11	8/9	3/3	47/50
Frequency of drug resistant HIV (n/n tested)	6/6	3/3	5/5	6/6	20/20	12/12	13/14	10/10	7/8	3/3	45/47
NRTI only	0	0	0	0	0/20	0	0	1	1	0	2
NNRTI only	0	0	0	0	0/20	3	1	0	0	0	4
NRTI + NNRTI	6	3	5	6	20/20	9	12	9	6	3	39
Global drug resistance n (%)	6/19 (31.6 %)	3/14 (23.1 %)	5/26 (19.2 %)	6/43 (13.9 %)	20/102 (19.6 %)	12/58 (20.7 %)	13/103 (12.6 %)	10/37 (27.0 %)	7/50 (14.0 %)	3/37 (8.1 %)	45/285 (15.8 %)
Resistance to 2 drugs of first line ART	4	3	3	2	12/20	2	6	5	2	0	15/45
Resistance to 3 drugs of first line ART	2	0	2	4	8/20	7	6	5	4	3	25/45
Cross-resistance to second line NNRTI											
ETV	1	0	1	1	3/20	3	3	3	1	1	11/45
RPV	3	1	3	6	13/20	9	8	6	5	3	31/45
Cross-resistance to other NRTIs											
ABC	2(I)	0	2	1	3/20	2	1	3	1	2	9/45
DDI	0	0	1	0	1/20	2	0	0	0	0	2/45
TDF	0	0	1	0	1/20	1	1	0	0	0	2/45

Seventy patients (18.1 %; CI95 14.5–22.2 %) had VL >1000 copies/ml; 20/102 (19.6 %; CI95 13.4–29.2 %) at M12 and 50/285 (17.5 %; CI95 13.5–22.4 %) at M24. Virological failure ranged from 13.9 to 31.6 % at M12 and from 8.1 to 22.4 % at M24. For 67 (95.7 %) patients, sequencing was successful and DRM were observed in 65 (97 %) of them; i.e. 20/20 at M12 and 45/47 at M24. Among the 65 drug resistant HIV strains, 59 were resistant to NRTIs and NNRTIs, two to NRTIs only and four to NNRTIs only. The global prevalence of drug resistance in the study population was thus at least 16.8 % (13.4–20.9 %, 95 % CI) (65/387), but 27 patients (6.9 %; 4.8–9.9 %, 95 % CI) were infected with HIV strains resistant to two of the three first-line ARVs and 32 (8.3 %; 5.9–11.4 %, 95 % CI) to all three first line ARVs.

As expected, the observed DRM were associated with the drugs used in first-line regimens (Table 2). M184 V selected by 3TC was the most frequent NRTI mutation, 56/65 (86.2 %). Frequently observed TAMs included M41L, D67 N/D, K70R, K219E/Q and T215Y/F. The K65R mutation was seen in two patients. One-third of the patients had at least 3 or more NRTI mutations and several patients were already predicted to be resistant to ABC (n = 12), DDI (n = 2) or tenofovir (TDF) (n = 3). Among NNRTI mutations, Y181C/Y and K103N were most frequently observed and 15 (23.1 %) patients accumulated at least three NNRTI mutations, with 14 (21.5 %) and 44 (67.7 %) that were predicted to be resistant to second line NNRTIs ETV and RPV, respectively.

**Table 2:** Drug resistance mutations to the first line antiretroviral drugs after 12 and 24 months on ART

Patient code	Months on ART	NNRTI mutations	NRTI mutations	Subtype/CRF	Accession
AN021	12	Y181V	M41LM, M184V, T215F	A3	KR047796
AN026	12	A98S, Y181C, G190A	M41L, A62V, M184V, K219N	CRF02_AG	KR047798
AN052	12	K103KN	M184V	CRF06_cpx	KR047805
AN058	12	V106A	A62AV, M184V	CRF02_AG	KR047807
AN059	12	K103N	M41L, M184V, T215Y	CRF02_AG	KR047808
AN074	12	K101E, G190A	M184V	CRF06_cpx	KR047810
AT407	12	K101E, G190A	M41L, D67N, K70R, V75IM, T215F, K219Q	URF	KR047840
AT411	12	Y181C, G190A	M184V	URF	KR047841
AT423	12	K103N	M184V	CRF06_cpx	KR047843
AT426	12	V90I, K101EQ, Y181C, G190S	K65R	CRF02_AG	KR047844
AT477	12	K103N	M184I	G	KR047850
KA305	12	Y181C	M41L, M184V	CRF06_cpx	KR047851
KA308	12	K103N, Y181C, H221Y	M184V, T215Y	CRF06_cpx	KR047852
KA348	12	Y181C	M184V	CRF06_cpx	KR047855
KA350	12	K101E, G190A	M41L, M184V	URF	KR047856
KA378	12	Y181C	M41L, M184V, L210W, T215Y	URF	KR047858
KA379	12	K103N, Y181C	M184I	CRF02_AG	KR047859
KP206	12	A98S, K103N, P225H	M184V	C	KR047826
KP218	12	K101EK, G190A	M184V	CRF06_cpx	KR047829
KP280	12	K103N	M184V	CRF02_AG	KR047837
AN008	24	V179I, Y181CY, G190AG	–	URF	KR047793
AN012	24	K103N	–	CRF02_AG	KR047794
AN019	24	Y181C, H221Y	M41L, V75I, M184V, T215F	CRF02_AG	KR047795
AN022	24	Y181C	M184V	URF	KR047797
AN028	24	A98S, Y181C	D67N, K70R, T215F, K219E	CRF02_AG	KR047799
AN032	24	Y181CY, G190AG, H221HY	–	URF	KR047800
AN041	24	K103N, Y181C	A62V, K65R, K70T, V75I, F116Y, Q151M, M184V	CRF02_AG	KR047801
AN043	24	K101E, Y181C, G190A	M184V, T215F	CRF02_AG	KR047802
AN048	24	Y181C	D67N, K70R, T215F, K219E	CRF02_AG	KR047803
AN049	24	K103N	M184V	CRF06_cpx	KR047804
AN054	24	K103N	M41L, E44D, L74I, M184V, L210W, T215Y	CRF02_AG	KR047806
AN068	24	Y181C, H221Y	M41LM, M184V, T215Y	CRF02_AG	KR047809
AT400	24	Y181C	K70KR, M184V	CRF02_AG	KR047838
AT403	24	–	M184V	A3	KR047839
AT420	24	K103N	M184V	CRF02_AG	KR047842
AT435	24	Y181C, H221Y	M41LM, D67DN, K70KR, T215Y, K219EK	CRF02_AG	KR047845
AT442	24	Y181C	M184V, T215FIST	CRF02_AG	KR047846
AT452	24	Y181C	M41L, M184V, T215Y	CRF02_AG	KR047847
AT456	24	K101E, G190A	M184V, T215F	CRF02_AG	KR047848
AT463	24	–	–	CRF02_AG	KR047849
KA319	24	A98S, K103N, Y181C	D67N, K70R, M184V, T215F, K219Q	URF	KR047853
KA344	24	K101E, G190A	M41L, D67DN, K70KR, M184V, T215Y	G	KR047854
KA366	24	K101E, Y181C, G190A	M41L, M184V, L210W, T215Y	URF	KR047857
KO100	24	Y181C	M41L, M184V, T215F	CRF06_cpx	KR047811
KO112	24	K103N, P225H	K70DEKN, M184V	CRF02_AG	KR047812
KO116	24	K103N	M184V	URF	KR047813
KO122	24	Y181C, H221Y	M41L, D67N, K70R, M184V, T215Y	G	KR047814
KO130	24	K103N, E138 K	M184V, T215F	CRF02_AG	KR047815
KO137	24	V179I, G190A, M230L	M184V, T215Y	A3	KR047816
KO148	24	K103N	M184V	CRF02_AG	KR047817
KO150	24	K103N, Y181C	M184V	CRF02_AG	KR047818
KO158	24	A98AG, K101E, Y181C	D67N, K70R, M184V, T215F, K219E	URF	KR047819
KO193	24	K103N	M184V	CRF02_AG	KR047820
KO195	24	Y181CY	–	CRF02_AG	KR047821
KO197	24	V90I, V179I, G190A	M184V	A3	KR047822
KO200	24	E138EG	–	URF	KR047823
KO203	24	Y181C, H221Y	M184V, T215Y,	CRF06_cpx	KR047824
KP202	24	Y181C, H221Y	M184V, T215Y	CRF02_AG	KR047825
KP210	24	G190S	M184V	CRF02_AG	KR047827
KP213	24	K103N	M184V, T215ST	CRF02_AG	KR047828
KP221	24	A98AS, K103N, E138Q,	M41L, V75I, M184 V, T215F	URF	KR047830
KP223	24	K103N, Y181C,H221Y	M41L, D67N, K70R, V75I, M184V, T215F, K219E,	CRF02_AG	KR047831
KP234	24	K103N	M41L, M184V	CRF06_cpx	KR047832
KP235	24	V90IV, A98AG, E138Q, V179T	M184V	CRF02_AG	KR047833
KP241	24	Y181V, H221Y	M41L, M184V, L210W, T215F	CRF02_AG	KR047834
KP243	24	K101E, G190A	M41L, D67N, M184V, L210W, T215Y	URF	KR047835
KP253	24	K103N	M184V	CRF02_AG	KR047836

### Discussion

Overall, we showed that 18 % of patients on ART for 12 or 24 months in semi-rural and rural ART clinics were on virological failure and almost all of them (97 %) were infected with drug resistant HIV-1 strains. These observations are high and close to what has been noticed in previous studies from Lomé, the capital city; for example in a survey conducted about 1 year earlier in 2010/2011 using the same cross-sectional approach, 19 % (124/642) of patients for 12 or 24 months on ART were infected with HIV drug resistant strains [2]. Our study confirms thus a high ART failure in Togo in general and which is higher than observed in other countries, when comparing with studies that used a similar approach [2]. Scale-up of ART started in 2007 in the capital city and was expanded to semi-rural areas in 2008. Between 2006 and 2012, the number of patients on ART increased from 6700 to 31,500 but tools to monitor patients did not follow this scale-up and training of medical personnel was insufficient to allow early detection of side_effects of certain ARVs, evaluate adherence or recognize rapidely decline of clinical status. In addition, the national program encountered problems with stock management resulting in ARV drug substitution with the same molecules, administered separately as individual pills instead as a fixed dosed combination, or even interruption of the treatment. It is known that non-adherence and treatment interruption may favor emergence of drug resistance.

Today, only very few studies reported observations on ART outcome from semi-rural or rural areas in resource limited settings, especially from Africa [2, 8, 9]. In Cameroon, 10 % of patients were infected with drug resistant HIV strains after a median of 12 months on ART in rural district hospitals at 50 to 150 km distance from Yaoundé, the capital city [10], which is close to rates observed in Yaoundé [2]. However, in a rural health center in Kolofata, at the extreme north of Cameroon at 1200 km from the capital city and with difficult connections, almost 30 % of patients on ART (median of 24 months) were resistant to ARV drugs [11]. Another report showed equal proportions of drug resistance in urban and rural areas, between 9.2 to 15.9 % after a median of 36 months on ART in Senegal, Mali and Guinea [12]. In rural and semi-rural settings in Gabon, 21 % of patients were resistant after a median of 33 months on ART [13]. In a rural clinic in Tanzania, rates of drug resistance were low and ranged from 4 to 8 % of patients after 1 or 2 years on ART, respectively [14]. Although, it is important to note that comparing results among the different studies mentioned above has to be taken with caution, because study design can differ as well as viral load capacities and techniques to identify virological failure.

Overall it seems that treatment outcome varies among countries, but within countries treatment outcome in semi-rural settings seem to be similar to those in urban settings except in extreme conditions. These observations are in line with the fact that national ART-programme management plays a role in treatment outcome in resource-limited countries [2]. It is important to note that we provided only information on the proportion of drug resistance in HIV infected patients who are still on ART and have no information on follow-up or mortality rates. Prospective studies where loss of follow-up are considered as treatment failures, would probable yield higher virological failure rates. Previous studies showed a higher mortality rate and loss of follow up in rural areas during the first 3 years [15].

Like in other reports on treatment outcome, several patients in our survey accumulated high numbers of mutations and developed also cross-resistance to potential second and/or third line drugs [16–18]. In addition these multi-drug resistant strains can also be transmitted and have a negative impact on future efficiency of first line regimens.

### Conclusions

The observations on ART treatment outcome in semi-rural areas show high failure rate but are close to those in Lomé, the capital city. Lowering the rates of drug resistance represents a challenge for the country. The first goals will be to identify factors associated with drug resistance.
